# Spatiotemporal Analysis of Hepatitis B Virus X Protein in Primary Human Hepatocytes

**DOI:** 10.1128/JVI.00248-19

**Published:** 2019-07-30

**Authors:** Dmytro Kornyeyev, Dhivya Ramakrishnan, Christian Voitenleitner, Christine M. Livingston, Weimei Xing, Magdeleine Hung, Hyock Joo Kwon, Simon P. Fletcher, Rudolf K. Beran

**Affiliations:** a Gilead Sciences, Foster City, California, USA; University of Southern California

**Keywords:** DDB1, HBx, Smc5/6 complex, hepatitis B virus

## Abstract

The structural maintenance of chromosomes 5/6 complex (Smc5/6) is a host restriction factor that suppresses hepatitis B virus (HBV) transcription. HBV counters this restriction by expressing the X protein (HBx), which redirects the host DNA damage-binding protein 1 (DDB1) E3 ubiquitin ligase to target Smc5/6 for degradation. HBx is an attractive therapeutic target for the treatment of chronic hepatitis B (CHB), but it is challenging to study this important viral protein in the context of natural infection due to the lack of a highly specific and sensitive HBx antibody. In this study, we developed a novel monoclonal antibody that enables detection of HBx protein in HBV-infected primary human hepatocytes (PHH) by Western blotting and immunofluorescence. Confocal imaging studies with this antibody demonstrated that HBx is predominantly located in the nucleus of HBV-infected PHH, where it exhibits a diffuse staining pattern. In contrast, a DDB1-binding-deficient HBx mutant was detected in both the cytoplasm and nucleus, suggesting that the DDB1 interaction plays an important role in the nuclear localization of HBx. Our study also revealed that HBx is expressed early after infection and has a short half-life (∼3 h) in HBV-infected PHH. In addition, we found that treatment with small interfering RNAs (siRNAs) that target DDB1 or HBx mRNA decreased HBx protein levels and led to the reappearance of Smc6 in the nuclei of HBV-infected PHH. Collectively, these studies provide the first spatiotemporal analysis of HBx in a natural infection system and also suggest that HBV transcriptional silencing by Smc5/6 can be restored by therapeutic targeting of HBx.

**IMPORTANCE** Hepatitis B virus X protein (HBx) is a promising drug target since it promotes the degradation of the host structural maintenance of chromosomes 5/6 complex (Smc5/6) that inhibits HBV transcription. To date, it has not been possible to study HBx in physiologically relevant cell culture systems due to the lack of a highly specific and selective HBx antibody. In this study, we developed a novel monoclonal HBx antibody and performed a spatiotemporal analysis of HBx in a natural infection system. This revealed that HBx localizes to the nucleus of infected cells, is expressed shortly after infection, and has a short half-life. In addition, we demonstrated that inhibiting HBx expression or function promotes the reappearance of Smc6 in the nucleus of infected cells. These data provide new insights into HBx and underscore its potential as a novel target for the treatment of chronic HBV infection.

## Abstract (precis)

Hepatitis B virus X protein (HBx) is a promising drug target since it promotes the degradation of the host structural maintenance of chromosomes 5/6 complex (Smc5/6) that inhibits HBV transcription. To date, it has not been possible to study HBx in physiologically relevant cell culture systems due to the lack of a highly specific and selective HBx antibody. In this study, we developed a novel monoclonal HBx antibody and performed a spatiotemporal analysis of HBx in a natural infection system. This revealed that HBx localizes to the nucleus of infected cells, is expressed shortly after infection, and has a short half-life. In addition, we demonstrated that inhibiting HBx expression or function promotes the reappearance of Smc6 in the nucleus of infected cells. These data provide new insights into HBx and underscore its potential as a novel target for the treatment of chronic HBV infection.

## INTRODUCTION

Approximately 240 million individuals worldwide are chronically infected with hepatitis B virus (HBV), and more than 650,000 people die each year due to HBV-associated liver diseases (1, 2). Current approved therapies for chronic hepatitis B (CHB) are limited to nucleos(t)ide analogs and interferon alpha, which reduce viral load and improve long-term outcome but rarely lead to cure (3). Therefore, there is an urgent need to develop novel antiviral therapies.

The HBV virion contains a 3.2-kb partially double-stranded DNA genome known as relaxed circular DNA (rcDNA). Following cell binding and entry, rcDNA is deposited in the nucleus and is repaired to form covalently closed circular DNA (cccDNA). This episome serves as the template for transcription of the viral RNAs. These viral RNAs are translated into various HBV proteins: the large, medium, and small envelope proteins (collectively HBsAg); E antigen (HBeAg); core; polymerase; and HBV X protein (HBx) (4).

HBx is a 17-kDa protein that plays an essential role in the HBV replication cycle (5, 6). It was recently determined that a key function of HBx is to promote the degradation of the cellular structural maintenance of chromosomes 5/6 complex (Smc5/6). Smc5/6 directly binds DNA (7, 8) and has been shown to topologically entrap DNA plasmids (9). Our previous work indicates that Smc5/6 directly binds to cccDNA and suppresses HBV transcription (10). However, HBV counters Smc5/6 restriction by expressing HBx, which binds and redirects the cellular DNA damage-binding protein 1 (DDB1)-containing E3 ubiquitin ligase to target Smc5/6 for ubiquitination and subsequent proteosomal degradation (10, 11).

HBx is a promising therapeutic target for the treatment of CHB because inhibition of this viral regulatory protein has the potential to transcriptionally silence cccDNA. However, it is challenging to study HBx due to experimental system and reagent limitations. For example, commonly used assay systems utilize dividing cells (whereas hepatocytes are quiescent) and express HBx at nonphysiological levels (e.g., overexpression by HBx plasmid transfection) (12). These experimental systems are often used out of necessity since it is difficult to study HBx in the context of natural infection due to the lack of a highly specific and sensitive HBx antibody (12). In recent years, the function of HBx has been characterized in more physiologically relevant systems, such as HBV-infected primary human hepatocytes (PHH) (6, 10, 11, 13). While this has yielded important insights into the role of HBx in the HBV replication cycle, there is still much to learn about this viral regulatory protein. In particular, the spatiotemporal characteristics of HBx in HBV-infected PHH have not been elucidated, and the impact of different therapeutic approaches on HBx expression and function has not been determined.

In the present study, we report the development of a novel anti-HBx monoclonal antibody (mAb). Using this new reagent, we determined the localization, half-life (*t*_1/2_), and expression kinetics of HBx in HBV-infected PHH. In addition, we used the HBx mAb to directly measure the effect of small interfering RNA (siRNA) treatment on HBx protein levels in this natural infection assay system.

## RESULTS

### Development and validation of a novel HBx antibody.

**FIG 1:**
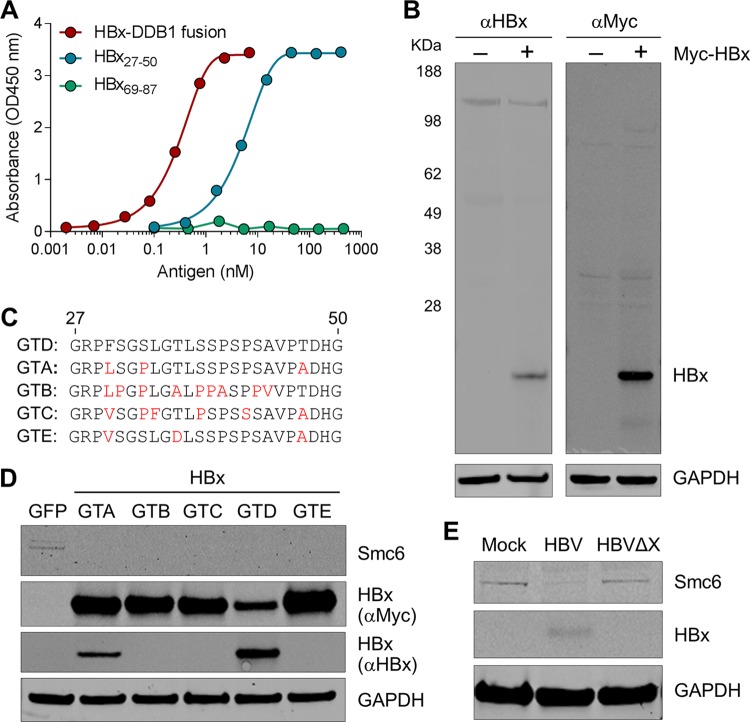
Validation of the anti-HBx mAb by an ELISA and Western blotting. (A) Binding of the HBx mAb to the recombinant HBx-DDB1 fusion protein, HBx_27–50_ peptide, and HBx_69–87_ peptide was evaluated by an ELISA. OD450 nm, optical density at 450 nm. (B) PHH were mock transduced or transduced with a lentivirus expressing Myc-tagged HBx. Cell lysates were analyzed by Western blotting at day 3 posttransduction. (C) Sequence of genotype D (GTD) HBx_27–50_ as well as the corresponding HBx sequences from genotypes A, B, C, and E. Residues in these HBx sequences that differ from those of genotype D HBx are indicated in red. (D) PHH were transduced with lentiviruses expressing GFP or HBx proteins of genotypes A to E. Cell lysates were analyzed by Western blotting at day 3 posttransduction. (E) PHH were mock infected or infected with HBV or HBVΔX. Cell lysates were analyzed by Western blotting at day 4 postinfection. A representative Western blot from 3 independent experiments with three independent PHH donors is shown.

In order to perform a spatiotemporal analysis of HBx in HBV-infected PHH, we first developed a highly specific and sensitive HBx mAb. Mice were immunized with a peptide corresponding to genotype D HBx residues 27 to 50 (HBx_27–50_), and the resulting hybridomas were evaluated by an enzyme-linked immunosorbent assay (ELISA) and confocal microscopy. The two hybridoma clones with the best signal-to-noise ratio (as measured by confocal imaging) were selected for monoclonal antibody production. The top mAb was chosen because it displayed superior sensitivity in a Western blot analysis. This anti-HBx mAb potently bound the HBx_27–50_ peptide but did not bind the HBx_69–87_ control peptide (Fig. 1A). This antibody also bound a recombinant HBx-DDB1 fusion protein (Fig. 1A), demonstrating that it can detect HBx when it is bound to this key interaction partner (10, 14).

**FIG 2:**
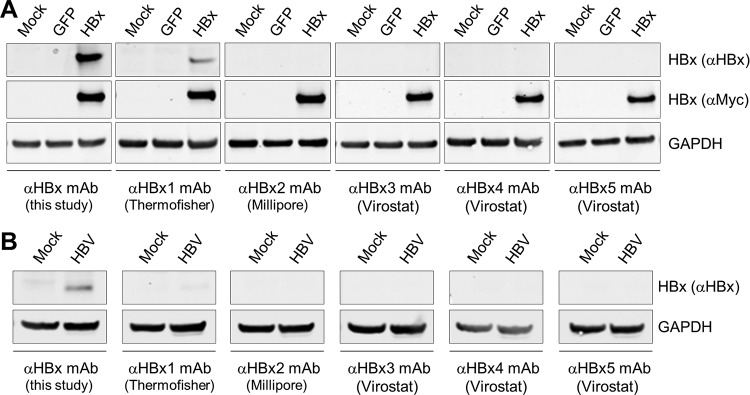
Comparison of the anti-HBx mAb with commercial anti-HBx antibodies by Western blotting. (A) PHH were mock transduced or transduced with lentiviruses expressing GFP or Myc-tagged HBx. Cell lysates were analyzed by Western blotting at day 5 posttransduction. (B) PHH were mock infected or infected with HBV. Cell lysates were analyzed by Western blotting at day 4 postinfection. Details of the commercial siRNAs are provided in Materials and Methods.

The HBx mAb was subsequently evaluated by Western blotting. The antibody detected a 17-kDa protein (the predicted size of Myc-HBx) in lysates from PHH transduced with a lentivirus expressing Myc-tagged HBx (genotype D) (Fig. 1B). This band was confirmed to be HBx using a Myc antibody (Fig. 1B). Consistent with substantial intergenotype sequence variation in the HBx_27–50_ sequence (Fig. 1C), we determined that the HBx mAb primarily recognizes genotype D HBx (Fig. 1D). Accordingly, genotype D HBV was used for all subsequent studies. We next demonstrated that the HBx mAb detects a 17-kDa band in lysates from HBV-infected PHH but not in lysates from mock-infected or HBx-negative virus (HBVΔX)-infected PHH (Fig. 1E). The same result was obtained using lysates from HepG2-NTCP cells that had been mock infected or infected with either HBV or HBVΔX (data not shown). In contrast, while one commercially available HBx antibody detected a 17-kDa band in lysates from PHH transduced with a lentivirus expressing Myc-tagged HBx (Fig. 2A), this antibody did not detect HBx in HBV-infected PHH (Fig. 2B). Collectively, these data demonstrate that the new anti-HBx mAb is selective for genotype D HBx and is able to detect physiologically relevant levels of HBx protein in the context of natural infection.

### HBx localizes to the nucleus of HBV-infected hepatocytes.

**FIG 3:**
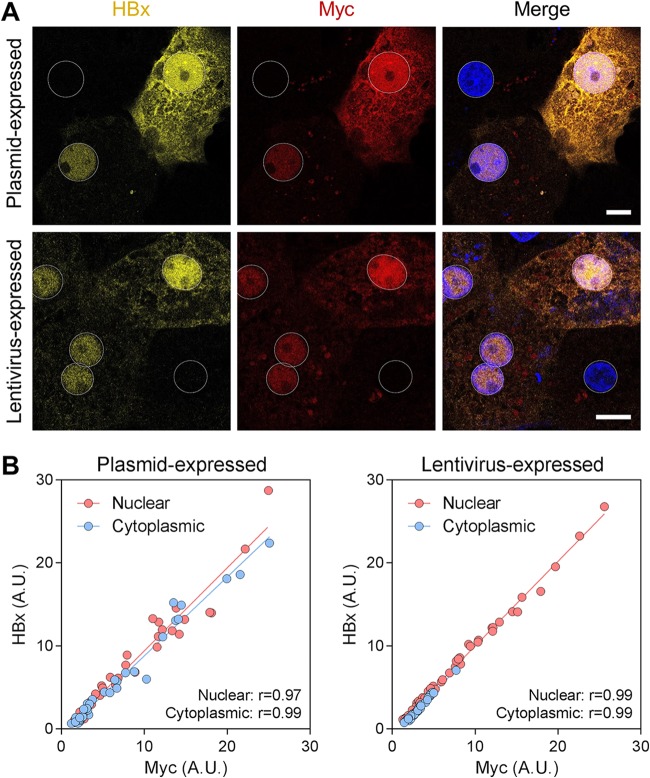
Validation of the anti-HBx mAb by immunofluorescence. (A, top) PHH were transfected with a plasmid expressing Myc-tagged HBx and analyzed by confocal microscopy at day 3 posttransfection. (Bottom) PHH were transduced with a lentivirus expressing Myc-tagged HBx and analyzed by confocal microscopy at day 5 posttransduction. PHH were stained for HBx (yellow) and Myc (red), and nuclei were stained with DAPI (blue). Bars, 10 μm. Nuclei are outlined by white dotted lines in all images. (B) Single-cell quantitation was performed to determine the HBx-specific fluorescence signal in the nucleus and the cytoplasm as measured by the HBx and Myc mAbs. The plots show the correlation between the HBx and Myc fluorescence signals in each cellular compartment. Each point represents an individual cell; ≥33 cells were analyzed in each cellular compartment. The linear association between the antibodies was calculated by Pearson correlation. *P* values for all correlations were <0.001. A.U., arbitrary units.

**FIG 4:**
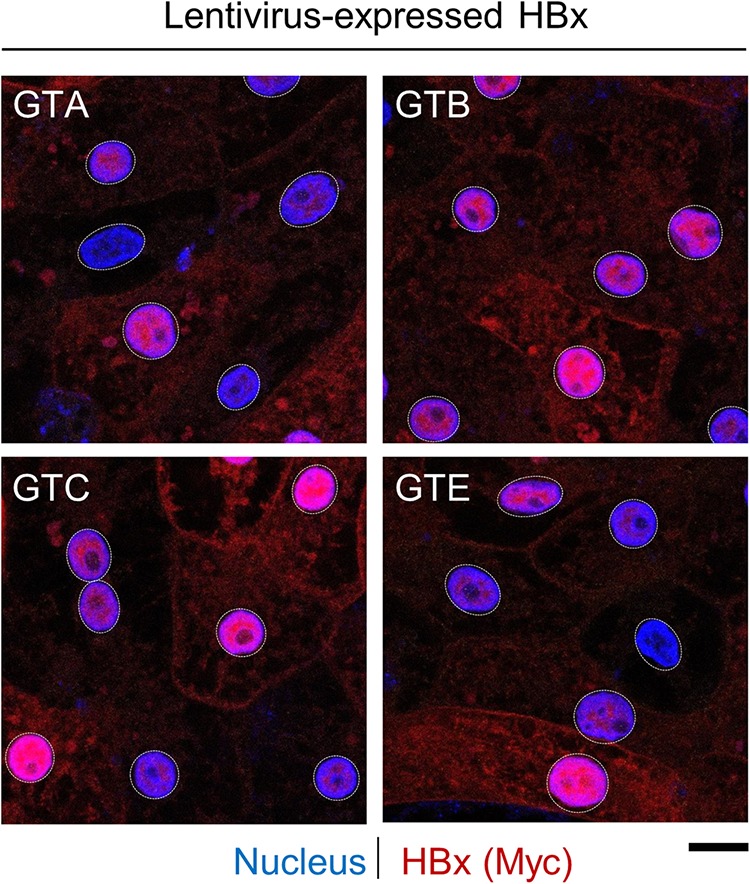
Comparable spatial distributions of HBx proteins from multiple HBV genotypes. PHH were transduced with lentiviruses expressing Myc-tagged HBx of genotype (GT) A, B, C, or E and analyzed by confocal microscopy at day 5 posttransduction. PHH were stained for Myc (red), and nuclei were stained with DAPI (blue). Bar, 10 μm. Nuclei are outlined by white dotted lines in all images.

After confirming the selectivity of the HBx mAb by ELISAs and Western blotting, this new reagent was evaluated by immunofluorescence. We demonstrated that the HBx mAb detected Myc-tagged HBx (genotype D) in PHH when it was expressed to supraphysiological levels by plasmid transfection and lentivirus transduction (Fig. 3A). HBx was detected in the nucleus of nearly all HBx-expressing PHH as well as in the cytoplasm of highly expressing cells. The spatial distribution of HBx was comparable to that of the Myc tag, and a close correlation between the fluorescence signals for the HBx and Myc antibodies was observed in both the nucleus and cytoplasm (Fig. 3B). Using the anti-Myc antibody, we confirmed that lentivirus-expressed Myc-tagged HBx proteins of genotypes A, B, C, and E display a spatial distribution similar to that of genotype D HBx (Fig. 4).

**FIG 5:**
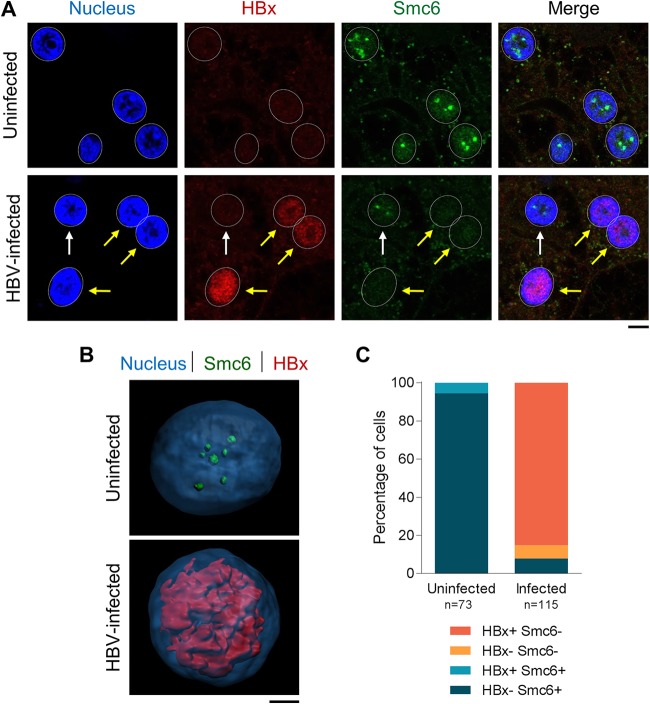
HBx localizes to the nucleus and inversely correlates with Smc6 in HBV-infected PHH. PHH were mock infected (uninfected) or infected with HBV and analyzed by confocal microscopy at day 13 postinfection. (A) PHH were stained for HBx (red) or Smc6 (green). Nuclei were stained with DAPI (blue). Yellow arrows indicate HBV-infected PHH (HBx-positive nuclei), and white arrows indicate uninfected cells. Representative confocal images from 8 independent experiments (8 to 14 days postinfection) with five independent PHH donors are shown. Bar, 5 μm. Nuclei are outlined by white dotted lines in all images. (B) Three-dimensional reconstruction of uninfected and HBV-infected PHH nuclei stained for HBx (red) and Smc6 (green). Nuclei were stained with DAPI (blue). Bar, 2 μm. (C) Single-cell quantitation of results from a representative experiment was performed, and each cell was classified by nuclear Smc6 and HBx status. The plot represents the number of cells in each population, expressed as a percentage of the total nuclei analyzed. The number of cells analyzed under each condition is displayed below the plot.

**FIG 6:**
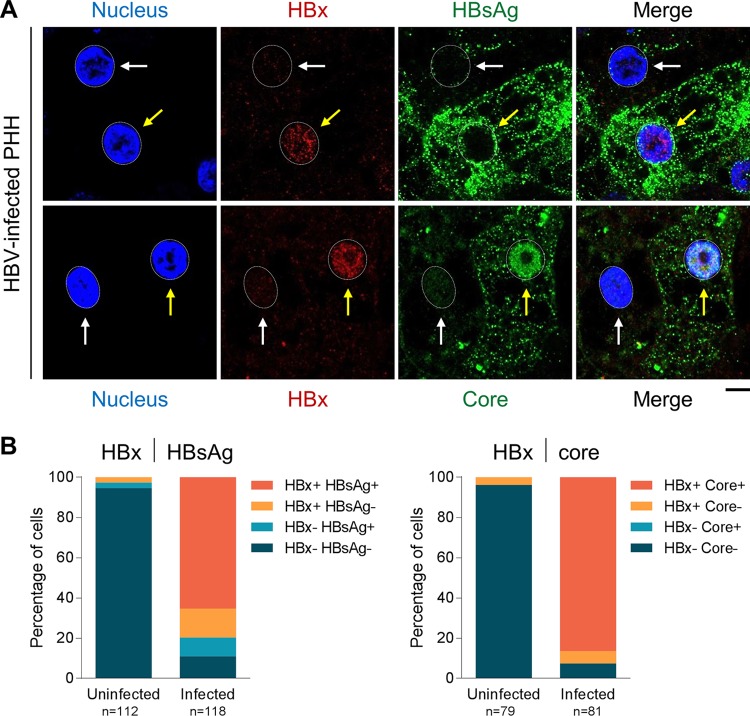
HBx positively correlates with HBsAg and HBV core in HBV-infected PHH. PHH were mock infected (uninfected) or infected with HBV and analyzed by confocal microscopy at day 13 postinfection. (A) PHH were stained for HBx (red), HBsAg (green) (top), or HBV core (green) (bottom). Nuclei were stained with DAPI (blue). Yellow arrows indicate HBV-infected PHH, and white arrows indicate uninfected cells. Representative confocal images from 2 independent experiments with two independent PHH donors are shown. Bar, 5 μm. Nuclei are outlined by white dotted lines in all images. (B) Single-cell quantitation of results of a representative experiment was performed, and each cell was defined by HBx and HBsAg status (left) or by HBx and nuclear core status (right). The plots represent the number of cells in each population, expressed as a percentage of the total nuclei analyzed. The number of cells analyzed under each condition is displayed below the plot.

After confirming the selectivity of the HBx mAb by immunofluorescence, we evaluated the spatial distribution of HBx in a natural infection system. In HBV-infected PHH, single-cell analysis revealed that HBx is predominantly located in the nucleus, where it exhibits a diffuse staining pattern (Fig. 5A and B). HBx was not detected in PHH that were mock infected or infected with an HBx-negative virus (HBVΔX) (Fig. 5A and data not shown). In contrast, none of the commercially available antibodies detected HBx in HBV-infected PHH (data not shown). Using the new HBx mAb, we demonstrated that the presence of HBx inversely correlated with the presence of Smc6 (Fig. 5A and B). Consistent with previous studies (10, 13), almost all uninfected PHH were Smc6 positive, whereas the majority of HBV-infected PHH were Smc6 negative and HBx positive (Fig. 5C). There was good concordance between the percentages of HBV-infected PHH that were Smc6 negative (92%) and those that were HBx positive (85%). HBx expression positively correlated with the presence of both HBsAg and HBV core in HBV-infected PHH (Fig. 6A and B), although there was better concordance between HBx and core than between HBx and HBsAg (Fig. 6B, compare sizes of dark orange bars). This is in line with previous studies comparing Smc6, core, and HBsAg levels in HBV-infected PHH (13).

### HBx interaction with DDB1 promotes nuclear localization.

**FIG 7:**
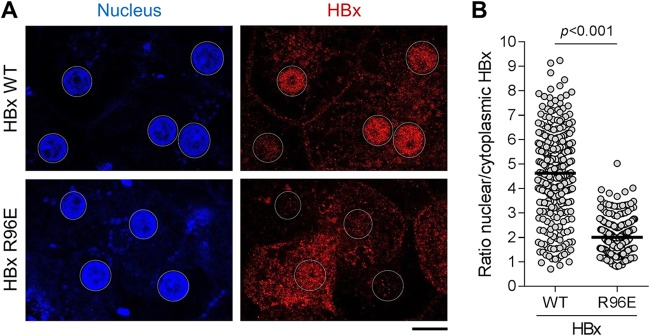
DDB1 binding alters HBx localization. PHH were transduced with a lentivirus expressing wild-type HBx or the DDB1-binding mutant HBx R96E and analyzed by confocal microscopy at day 3 posttransduction. (A) PHH were stained for HBx (red), and nuclei were stained with DAPI (blue). Representative confocal images from 3 independent experiments with two independent PHH donors are shown. Bar, 10 μm. Nuclei are outlined by white dotted lines in all images. (B) Single-cell quantitation was performed to determine the HBx-specific fluorescence signal in the nucleus and the cytoplasm of transduced cells. The plot shows the ratio of nuclear to cytoplasmic HBx. Each point represents an individual cell, and the line represents the mean. Data from 3 independent experiments with two independent PHH donors are shown; ≥56 cells were analyzed under each condition in each experiment. Nonexpressing cells were excluded from the analysis after they were identified using cutoff values based on the nuclear HBx-specific fluorescence signal in PHH transduced with a control (GFP-only) lentivirus. Statistical significance was calculated by a two-tailed *t* test.

We have previously demonstrated that Smc5/6 localizes with ND10 in the nuclei of uninfected PHH and that HBV infection does not induce the degradation of the ND10 structural components PML and Sp100 (13). The diffuse nuclear staining pattern of HBx indicates that it does not predominantly localize to these intranuclear structures. We therefore attempted to determine whether HBx instead colocalizes with DDB1 in HBV-infected PHH. Unfortunately, we were not successful in identifying a DDB1-selective antibody that was suitable for immunofluorescence. As an alternative approach, we compared the spatial distribution of lentivirus-expressed wild-type (WT) HBx with that of the DDB1-binding mutant HBx R96E (genotype D in both cases) (14, 15). Consistent with the spatial distribution of lentivirus-expressed HBx (genotypes A to E) and HBx expressed in the context of natural infection (genotype D), lentivirus-expressed WT HBx predominantly localized to the nucleus of PHH, particularly in cells with lower levels of HBx expression (Fig. 7A). Conversely, HBx R96E was prominently detected in the cytoplasm as well as the nucleus (Fig. 7A). Quantitative single-cell analysis demonstrated that the HBx R96E mutant has a significantly lower ratio of nuclear to cytoplasmic signals than wild-type HBx (mean of 2.0 versus 4.6, respectively), confirming that the DDB1-binding mutant is more evenly distributed between these cellular compartments (Fig. 7B). Taken together, these data suggest that the interaction with DDB1 plays an important role in the nuclear localization of HBx.

### HBx is expressed early after HBV infection.

**FIG 8:**
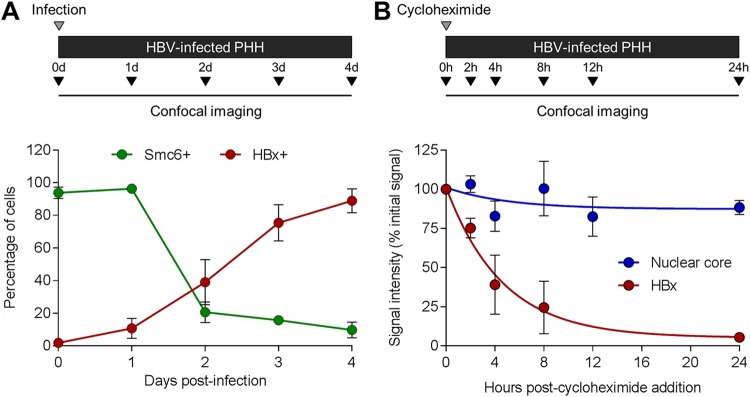
HBx is expressed shortly after HBV infection and has a short half-life in PHH. (A) PHH were infected with HBV, and nuclear HBx and Smc6 mean fluorescence intensity levels were measured on the indicated days postinfection by confocal microscopy. PHH were stained as described in the legend of Fig. 5A, and single-cell quantitation was performed as described in the legend of Fig. 5C. HBV core and Smc6 status of HBV-infected PHH was determined using background levels calculated from time-matched mock-infected controls. Data from 3 independent experiments with two independent PHH donors are shown; circles indicate mean levels, and the error bars represent the SEM. (B) PHH were infected with HBV and 5 days later treated with 100 μg/ml cycloheximide. Nuclear HBx and HBV core mean fluorescence intensity levels were measured at the indicated times posttreatment by confocal microscopy, and the data were fit to a first-order exponential decay equation. Data from 3 independent experiments with three independent PHH donors are shown; circles indicate mean levels (expressed as a percentage of the value for the pretreatment control), and the error bars represent the SEM.

Our previous study indicated that HBx is expressed early after HBV infection using Smc6 degradation as a functional readout of HBx expression (13). However, we were unable to directly measure the kinetics of HBx expression in these previous experiments. We therefore performed a series of imaging studies to characterize the kinetics of HBx expression in HBV-infected PHH. This revealed that a few HBx-positive cells could be detected at 24 h postinfection, with the percentage of HBx-positive cells increasing rapidly thereafter (Fig. 8A). In line with our previous study, the percentage of Smc6-positive cells sharply declined by day 2 postinfection (Fig. 8A). Interestingly, there were more Smc6-negative cells (80%) than HBx-positive cells (40%) at day 2 postinfection, whereas there was good concordance between these measures on days 3 and 4 postinfection. These data suggest that very low levels of HBx (i.e., below the detection limit of this assay) are sufficient to target Smc6 for degradation.

### HBx has a short half-life in HBV-infected PHH.

The half-life of HBx has been evaluated in various experimental systems (16, 17) but never in the context of natural infection. In order to do so, the levels of HBx and core protein in the nucleus of HBV-infected PHH were measured by confocal microscopy at various times after treatment with the translation inhibitor cycloheximide. This revealed that HBx has a half-life of approximately 3 h in HBV-infected PHH (Fig. 8B). In contrast, nuclear HBV core was extremely stable, with a half-life of >24 h (Fig. 8B).

### Reducing HBV mRNA or inhibiting HBx function decreases HBx protein levels and leads to reappearance of Smc6 in HBV-infected hepatocytes.

**FIG 9:**
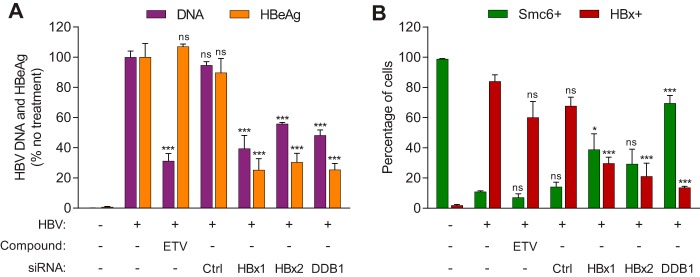
Inhibition of HBx and reappearance of Smc6 after treatment of HBV-infected PHH with siRNA that targets HBx or DDB1. HBV-infected PHH were transfected with siRNA to the indicated gene or a nontargeting control siRNA (Ctrl) or mock transfected on day 6 postinfection or were treated with 1 nM entecavir (ETV) from days 6 to 21 postinfection. Mock-infected (uninfected) PHH were included as a control. (A) Plot showing extracellular HBV DNA and HBeAg at day 21 postinfection. Data are expressed as a percentage of the value for the no-treatment control; the bar height indicates the mean of data from 3 independent experiments with three independent PHH donors, and the error bars represent the SEM. (B) PHH were analyzed by confocal microscopy at day 21 postinfection. PHH were stained as described in the legend of Fig. 5A, and single-cell quantitation was performed as described in the legend of Fig. 5C. Data from 3 independent experiments with three independent PHH donors are shown; the bar height indicates the mean, and the error bars represent the SEM. At least 92 nuclei were analyzed under each condition. Statistical significance relative to the no-treatment control was calculated by one-way ANOVA with Dunnett’s multiple-comparison correction. *, *P* < 0.05; ***, *P* < 0.001; ns, not significant (*P* > 0.05). Data from uninfected PHH were not included in the statistical analysis.

A number of siRNAs are currently in preclinical and clinical development for the treatment of CHB (18, 19). We therefore determined if HBx protein levels could be reduced by RNA interference and whether this would lead to the reappearance of the Smc5/6 complex and silencing of cccDNA transcription. HBV-infected PHH were either treated with the nucleoside analogue entecavir (ETV) or transfected with a nontargeting control siRNA (siCtrl), an siRNA targeting the HBx open reading frame (siHBx1 or siHBx2), or an siRNA targeting the HBx binding partner DDB1 (siDDB1). The siRNAs were transfected into HBV-infected PHH at day 6 postinfection, by which time Smc6 had been degraded in the majority of infected cells (Fig. 8A) (13). As expected, ETV significantly reduced extracellular HBV DNA levels but not HBeAg levels, and siCtrl had no antiviral activity (Fig. 9A). Neither ETV nor siCtrl significantly reduced the percentage of HBx-positive cells or increased the percentage of Smc6-positive cells (Fig. 9B). In contrast, treatment with siHBx1 or siHBx2 significantly reduced extracellular HBV DNA and HBeAg levels (Fig. 9A) as well as the percentage of HBx-positive cells (Fig. 9B). However, while ≤30% cells were still HBx positive after treatment with these siRNAs, ≤40% cells became Smc6 positive. Thus, there was a sizeable population of PHH (∼30% for siHBx1 and 50% for siHBx2) which remained Smc6 negative after siHBx treatment but had no detectable HBx (i.e., below the detection limit of this assay). This observation is in line with the viral kinetics data suggesting that low levels of HBx are sufficient to target Smc5/6 for degradation (Fig. 8A).

As previously reported (10), inhibiting HBx function with siDDB1 significantly reduced extracellular HBV DNA and HBeAg levels (Fig. 9A). This siRNA also significantly reduced the number of HBx-positive cells (Fig. 9B). In contrast to siHBx1 and siHBx2, the majority of cells (70%) became Smc6 positive after siDDB1 treatment (Fig. 9B). Collectively, these data demonstrate that although siRNAs targeting HBx can significantly decrease HBx levels in HBV-infected PHH, restoration of Smc5/6 requires very potent HBx reduction.

## DISCUSSION

HBx research is often controversial due to a reliance on experimental systems with the potential to generate physiologically irrelevant phenotypes (12). In particular, differences in cell culture systems and HBx expression levels have led to conflicting reports regarding the spatiotemporal characteristics of HBx. We therefore developed a novel HBx mAb and used it to characterize HBx in HBV-infected PHH. By doing so, the present study provides the first spatiotemporal analysis of HBx in the context of a natural infection system.

An important finding from this study is that HBx is predominantly located in the nucleus of HBV-infected PHH. This observation is consistent with a key function of HBx being targeting of nuclear Smc5/6 for degradation (10, 11, 13). Our data are in line with previous studies which concluded that nuclear localization of HBx is required for its function (20, 21). Conversely, HBx has been reported to localize to the cytosol and to associate with mitochondria (22–25). It has been proposed that the subcellular localization of HBx is influenced by its abundance (25, 26), suggesting that differences in expression levels may account for the discrepancies between these studies. Our data with PHH are consistent with the spatial distribution of HBx being a function of the expression level. We found that HBx is detected in both the nucleus and cytoplasm of cells with high HBx levels (e.g., plasmid-transfected PHH), whereas HBx is predominantly located in the nucleus of cells with low-level HBx expression (e.g., HBV-infected PHH). Since HBx does not possess a nuclear localization signal, it has been proposed that the nuclear accumulation of HBx depends on interaction with a cellular protein (27, 28). This hypothesis would explain why the capacity for nuclear import or retention of HBx is limited. Notably, the cytoplasmic concentration of HBx is increased when DDB1 binding is reduced (e.g., by the R96E point mutation). These data therefore suggest that the cytoplasmic localization of HBx, which occurs in the context of high-level HBx expression, may be a consequence of saturation of the HBx-DDB1 interaction.

The HBx mAb described in the present study was raised against genotype D HBx residues 27 to 50 (HBx_27–50_). This region is largely unstructured and is not required for HBx function *in vitro* (29–31) but has substantial intergenotype sequence variation. Accordingly, targeting HBx_27–50_ has key advantages but some important limitations. The main benefit of the mAb recognizing a nonconserved and nonessential epitope is that it minimizes the risk that HBx-interacting partners affect antibody recognition. Importantly, such a mAb should retain the ability to detect HBx when it is bound to DDB1, which is a key interaction partner of HBx (10, 14). In the absence of a DDB1-selective antibody suitable for immunofluorescence, it is challenging to directly confirm that the HBx mAb detects HBx associated with endogenous DDB1 in PHH. However, we demonstrated that the antibody can bind a purified recombinant HBx-DDB1 fusion protein (Fig. 1A). Importantly, HBx is soluble and exhibits stoichiometric zinc binding only when expressed in the presence of DDB1, suggesting that HBx in the fusion protein adopts a physiologically relevant conformation (29). Furthermore, the HBx-DDB1 fusion protein expressed in PHH promotes Smc6 degradation and rescues HBVΔX transcription (29). Another advantage of using a mAb that recognizes the N-terminal region of HBx is that it will not detect a short HBx protein (HBx_79–154_) which may be expressed in HBV-infected cells (32). This is apposite because this shorter HBx protein is likely not functional in the context of natural infection *in vitro* (29).

The main disadvantage of using an antibody raised to genotype D HBx_27–50_ is that it is primarily selective for this one HBV genotype. Accordingly, all natural infection studies with this mAb were limited to HBV genotype D. However, our data suggest that the predominant nuclear localization of HBx that we observed in these studies is relevant to other genotypes. First, we demonstrated that the subcellular localizations of lentivirus-expressed HBx proteins from genotypes A to E are comparable in PHH and that the spatial distribution of lentivirus-expressed genotype D HBx in PHH is similar to that of HBx expressed in the context of natural infection. These data are consistent with the observation that HBx proteins from HBV genotypes A to E all promote the degradation of nuclear Smc6 (Fig. 1D). Second, our data indicate that the DDB1 interaction plays an important role in the nuclear localization of HBx. It was previously shown that X proteins from various mammalian hepadnaviruses bind DDB1 (33), suggesting that nuclear localization is characteristic of mammalian hepadnavirus X proteins when expressed at physiologically relevant levels. Collectively, these data indicate that our findings with genotype D HBx can be extended to HBx proteins from other HBV genotypes.

Another notable finding from this study is that the half-life of HBx is substantially shorter than that of nuclear HBV core in HBV-infected PHH (∼3 h versus >24 h, respectively) and shorter than those of many proteins in PHH (34). These data are an important confirmation of the results from previous overexpression studies (16, 17). It has been reported that HBx is stabilized by the interaction with DDB1 in Chang cells (35). Our observation that the cellular localization of HBx is influenced by the interaction with DDB1 provides a potential explanation for why DDB1 binding alters HBx stability. It was also previously suggested that HBx expression is limited to the early stages of infection (36). In light of its short half-life, the detection of HBx in HBV-infected PHH both after the initial stage of infection and at steady state (13 to 21 days postinfection) indicates that HBx is continuously expressed after infection. This is consistent with the requirement for HBx for both the initiation and maintenance of HBV replication after infection (6).

A previous study suggested that HBx is expressed early after HBV infection using Smc6 degradation as a marker for HBx expression (13). In the present study, we confirmed that HBx is expressed shortly after HBV infection of PHH. However, HBx is required to alleviate transcriptional suppression by Smc5/6, which raises the question of how expression of HBx itself is induced. One possibility is that there is a lag between the initiation of HBV transcription and the detection of cccDNA by Smc5/6. This is in line with a previous study in which we observed transient, low-level production of viral antigens in the first few days after infection of PHH with HBVΔX (13). An alternative (but not necessarily mutually exclusive) explanation for how HBx is initially expressed is provided by a recent study which detected HBx RNA by transcriptome sequencing in cell culture-derived virus preparations as well as CHB patient plasma (13). This led to the hypothesis that HBx is initially expressed by delivery of extracellular HBx RNA (or spliced HBV RNA) into HBV-infected cells (13, 37). However, much work remains to determine if extracellular HBV RNA plays an important role in the viral replication cycle. Work is ongoing to determine the mechanistic basis of Smc5/6 recognition of cccDNA and other episomal templates and to further characterize extracellular HBV RNA.

Inhibiting HBx by RNA interference is a promising therapeutic strategy since restoration of Smc5/6 should lead to transcriptional silencing of cccDNA. In addition, since the HBx mRNA sequence is coterminal with all other HBV RNAs, an siRNA trigger in HBx mRNA should target all HBV RNAs for degradation. However, the overlap of the HBV RNA sequences precludes the selective detection of HBx mRNA by reverse transcription-quantitative PCR. Moreover, it is challenging to measure HBx mRNA levels by Northern blotting (13). Therefore, while it is expected that HBx siRNA treatment would reduce HBx levels, to our knowledge, it has never been directly demonstrated. In the present study, we confirmed that siRNAs targeting HBx can significantly decrease HBx levels in HBV-infected PHH. Most importantly, we determined that restoration of Smc5/6 requires very potent HBx reduction. To date, selection of HBx siRNA triggers has typically relied on measurement of surrogate viral endpoints (e.g., viral DNA and HBsAg) to infer anti-HBx activity. However, data from the present study indicate that neither a reduction of standard HBV replication endpoints nor a decrease in intracellular HBx levels is a good predictor of HBx functional inhibition by siRNA triggers. Instead, our data suggest that restoration of Smc6 levels in HBV-infected hepatocytes is the most important criterion for identifying effective HBx siRNA triggers. As an alternative approach, inhibiting HBx function, modeled in the present study by siDDB1 treatment, may be an efficient way to induce cccDNA transcriptional silencing by the Smc5/6 complex. Indeed, inhibiting the HBx-DDB1 interaction is a promising therapeutic strategy because it is expected to alter HBx cellular localization and reduce HBx stability (35), in addition to inhibiting HBx function.

In summary, we have developed a novel anti-HBx antibody that is an important addition to the HBV reagent armamentarium. This mAb has yielded new insights into virus-host interactions and also has utility for evaluating new therapeutic agents.

## MATERIALS AND METHODS

### Reagents.

The HepAD38 and the HepG2-H1.3x^−^ stable cell lines have been previously described (6, 38) and were obtained from Avid Therapeutics (Philadelphia, PA) and Ulrike Protzer, respectively. Cryopreserved PHH isolated from livers of deceased donors were purchased from Life Technologies (Grand Island, NY). Consent was obtained from the donor or the donor’s legal next of kin for use of these samples and their derivatives for research purposes using institutional review board (IRB)-approved authorizations. Rabbit polyclonal anti-HBV core (Agilent, Santa Clara, CA), rabbit monoclonal anti-HBV core (clone 366-2) (Gilead Sciences, Foster City, CA), rabbit polyclonal anti-Smc6 (MilliporeSigma, St. Louis, MO), mouse monoclonal anti-Smc6 (clone 2E7/M01) (Abgent, San Diego, CA), rabbit monoclonal anti-HBsAg (ViroStat, Westbrook, ME), mouse monoclonal anti-Myc (clone 9E10) (LifeSpan Biosciences, Inc., Seattle, WA), mouse monoclonal anti-Myc antibody directly conjugated to DyLight 650 (clone Myc.A7) (Thermo Fisher Scientific, Waltham, MA), and rabbit anti-glyceraldehyde-3-phosphate dehydrogenase (GAPDH) (clone 14C10) (Cell Signaling, Danvers, MA) were used for Western blotting and confocal imaging. Various commercial mouse monoclonal anti-HBx antibodies were used for Western blotting and confocal imaging: clone X36C (referred to as anti-HBx1; Thermo Fisher Scientific), clone 227 (anti-HBx2; MilliporeSigma), and clones 1882, 1883, and 1884 (anti-HBx3, anti-HBx4, and anti-HBx5, respectively; ViroStat). Alhydrogel (ALD; InvivoGen, San Diego, CA) and muramyl dipeptide (MDP; MilliporeSigma) were used as an adjuvant for mouse immunizations. Entecavir was purchased from Selleck Chem (Houston, TX). An HBx-expressing plasmid was produced by GenScript (Piscataway, NJ). Genotype D HBx was expressed under the control of a cytomegalovirus (CMV) promoter from pcDNA3.1^+^ (Thermo Fisher Scientific). All lentiviruses were produced by System Biosciences (Palo Alto, CA). The HBx sequences are provided in Table 1. HBx expressed from both plasmids and lentiviruses included an N-terminal 3×Myc tag. The recombinant His-HBx-DDB1 fusion protein was expressed and purified as previously described (29). All siRNAs were obtained from Dharmacon (Boulder, CO). The HBV (siHBx1 and siHBx2), DDB1 (siDDB1), and control (siCtrl1) siRNAs have been previously described (10, 39). In the original description, siHBx1 was referred to as “HBx-1,” and siHBx2 was referred to as “HBx-3” (39). The primers and probes for quantitative PCR (qPCR) were synthesized by IDT (Coralville, IA) (HBV) and Life Technologies (β-actin).

**TABLE 1:**
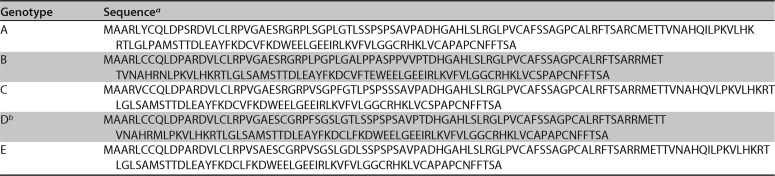
HBx sequences used in this study

a Consensus sequences for genotypes A, B, C, and E were generated as described in Materials and Methods.

b GenBank accession number AUG90794.

### Monoclonal antibody production.

Mouse monoclonal anti-HBx antibodies were generated by Antibody Solutions (Sunnyvale, CA). Five peptide sequences approximately 20 residues long spanning various regions of HBx (excluding the HBx-DDB1 interaction region) were analyzed for solubility, secondary structure, as well as antigenic potential using established methods (40, 41). Two of these HBx peptides were individually conjugated to bovine serum albumin (BSA), and three NZB/W, three CD-1, and three BALB/c mice were immunized with 10 μg of a peptide-BSA conjugate plus 1 μg of adjuvant (ALD and MDP in a 1:1 ratio). The animal protocol and all procedures involving mice were approved by the Institutional Animal Care and Use Committee and adhered to those in the *Guide for the Care and Use of Laboratory Animals* (46) and of the American Veterinary Medical Association. Biweekly injections were performed using a “rapid” immunization protocol developed by Antibody Solutions. The HBx AD38 (genotype D) peptide spanning residues 27 to 50 (HBx_27–50_) was immunogenic in CD-1 mice, and the resulting hybridomas were evaluated for binding to the recombinant HBx-DDB1 fusion protein by an ELISA. The top 50 candidates were further evaluated by confocal imaging of HBV-infected PHH. Monoclonal antibodies (mAbs) were developed from the two most selective and sensitive hybridoma clones (M14 and M19). The top antibody clone (M19) was selected after Western blot analysis of lentivirus-expressed HBx in PHH.

To generate a rabbit anti-HBx antibody, the variable domains of the heavy (VH) and light (VL) chains of the mouse HBx antibody were PCR amplified with primers encompassing 15-nucleotide overhangs to the rabbit immunoglobulin heavy and kappa constant region coding sequences. The VH and VL PCR bands were gel extracted using the QIAquick gel extraction kit (Qiagen, Valencia, CA) and cloned into the pcDNA3.1 expression vector with the rabbit immunoglobulin heavy and kappa constant PCR bands, respectively, using an In-Fusion HD cloning kit (TaKaRa Bio USA, Mountain View, CA). The heavy and light chain expression plasmids were then transfected into Expi293F cells according to the manufacturer’s protocol (Thermo Fisher Scientific). At 4 days posttransfection, the cells were pelleted by centrifugation, and the medium was collected. The rabbit anti-HBx antibody was purified from the medium by protein A chromatography.

### HBx ELISA.

Antibody candidates were screened by ELISAs. Briefly, sulfhydryl plates were coated with 36 ng/ml recombinant HBx-DDB1 fusion protein, blocked, and then incubated with 50 μl of an undiluted hybridoma supernatant. The signal was detected colorimetrically with a goat anti-mouse–horseradish peroxidase (HRP) antibody (Jackson Immuno Research, West Grove, PA) and by incubation with a 3,3',5,5'-tetramethylbenzidine (TMB) substrate solution (MossBio, Pasadena, MA). Signal strength was measured as the absorbance at 450 nm. ELISA profiling of mAbs was performed using 100 ng/ml antibody with various concentrations of the recombinant HBx-DDB1 fusion protein or BSA-conjugated HBx peptide.

### HBV virion production and PHH infection, transduction, and transfection.

Production of wild-type HBV virions (genotype D) from HepAD38 cells and HBVΔX virions (genotype D) from the HepG2-H1.3x^−^ stable cell line was performed as previously described (10, 13). PHH were infected with HBV or HBVΔX (1,000 genomic equivalents/cell) or transduced with lentiviruses as described previously (13). For plasmid transfections, 1.5 million PHH were plated per well in a 6-well plate, incubated overnight, and then transfected with 0.5 μg DNA using Lipofectamine 3000 (Thermo Fisher Scientific) according to the manufacturer’s instructions. For the RNA interference studies, PHH were transfected with 25 nM siRNA using Lipofectamine RNAiMax (Thermo Fisher Scientific) according to the manufacturer’s instructions.

### Western blotting.

Western blotting was performed as previously described (10). Briefly, PHH were transduced with a lentivirus expressing 3×Myc-HBx (HBV genotype A, B, C, D, or E), infected with wild-type HBV or HBVΔX, or mock infected. On the indicated day posttransduction or postinfection, lysates were collected by adding 0.25 ml 1× cell lysis buffer (Cell Signaling) combined with a protease inhibitor cocktail (Thermo Fisher Scientific) and scraping to remove the cells from the plates. Membranes were probed with a 1:250 dilution of mouse monoclonal anti-Smc6 (Abgent), a 1:1,000 dilution of mouse monoclonal c-Myc (LifeSpan Biosciences), a 1:250 dilution of mouse monoclonal anti-HBx mAb (15-μg/ml final concentration), and a 1:2,000 dilution of rabbit monoclonal anti-GAPDH (Cell Signaling). Commercially available anti-HBx antibodies were tested at the concentrations recommended by the manufacturers. Anti-HBx antibodies from Thermo Fisher Scientific and Millipore were used at 1 μg/ml, and the anti-HBx antibodies from ViroStat were used at 2 μg/ml. IRDye 680RD goat anti-rabbit or IRDye 800CW goat anti-mouse IgG (Li-Cor, Lincoln, NE) at a 1:5,000 dilution was used as the secondary antibody. Blots were visualized using an Odyssey infrared imaging system (Li-Cor).

### Confocal microscopy.

PHH were seeded onto glass coverslips (BioCoat poly-d-lysine/laminin, 12 mm; Corning, Corning, NY) in 12-well Corning Cellbind plates and then infected, transduced, or transfected on the following day. On the indicated day postinfection, posttransduction, or posttransfection, cells were fixed for 10 min using Perfusion fixative reagent (Thermo Fisher Scientific) and washed three times in Dulbecco’s phosphate-buffered salt solution (DPBS) (Corning). All steps of the immunostaining protocol were performed at room temperature. Cells were then permeabilized in 0.3% Triton X-100 (MilliporeSigma) for 15 min, followed by blocking in DPBS with 3% BSA (MilliporeSigma) and 10% HyClone fetal bovine serum (FBS) (MilliporeSigma) for 60 min. The final concentrations of primary antibodies were 0.2 μg/ml for mouse anti-HBx mAb, 1 μg/ml for rabbit anti-HBx mAb, 1 μg/ml for rabbit anti-Smc6 (MilliporeSigma), 1 μg/ml for mouse anti-Smc6 (Abgent), a 1:100 dilution for mouse anti-Myc antibody directly conjugated to DyLight 650 (Thermo Fisher Scientific), a 1:500 dilution for rabbit anti-HBsAg antibody (ViroStat), 0.5 μg/ml for rabbit anti-HBV core antibody (Gilead Sciences), and a 1:1,600 dilution for rabbit anti-HBV core antibody (B0586; DAKO, Denmark). Secondary antibodies conjugated with either Alexa Fluor 488 (goat anti-rabbit IgG; Thermo Fisher Scientific), Alexa Fluor 555 (goat anti-rabbit IgG; Thermo Fisher Scientific), or Alexa Fluor 647 [donkey anti-mouse IgG(H+L); Thermo Fisher Scientific] were used at 2 μg/ml. All antibodies were diluted in DPBS containing 1.5% BSA. Primary and secondary antibodies were applied for 90 min and 60 min, respectively. The coverslips with stained cells were mounted onto glass microscopic slides (VWR International, Radnor, PA) with a drop of ProLong gold antifade reagent containing DAPI (4′,6-diamidino-2-phenylindole) (Thermo Fisher Scientific). The samples were imaged with a Leica SP8 confocal laser scanning microscope (Leica Microsystems, Inc., Wetzlar, Germany). All images within each sample set were captured using identical instrument settings. Acquisition was performed in two sequences to minimize bleed-through artifacts. During the first sequence, DAPI was excited at 405 nm with a UV laser and detected at 415 to 494 nm, while Alexa Fluor 647 was excited at 647 nm and detected at 657 to 800 nm. During the second sequence, Alexa Fluor 488 was excited at 499 nm and detected at 504 to 648 nm. Lentivirus-expressed HBx was stained with Alexa Fluor 555 due to the presence of green fluorescent protein (GFP) in the construct. In this case, detection was performed in three sequences by adding a sequence to measure the Alexa Fluor 555 signal by exciting the fluorophore at 555 nm and detecting fluorescence at 565 to 640 nm.

The images were analyzed with ImageJ 1.51u (NIH, Bethesda, MD). Batch processing to identify cells positive for HBx, Smc6, nuclear HBV core, or HBsAg was performed with Imaris 9.1.2 image analysis software (Bitplane, Belfast, UK), using an algorithm based on the Cell Detection function (smooth filter width, 0.0401 μm; diameter of seed points, 5 μm). The HBx, HBV core, and HBsAg background was calculated as the mean plus 2 times the standard deviations of the background signal in mock-infected PHH. The Smc6 background was calculated as the mean minus 2 times the standard deviations of the Smc6 level in mock-infected PHH. Values above these limits were considered positive, and values below were considered negative. For HBx, HBV core, and Smc6 proteins in HBV-infected PHH, these measurements were restricted to the nucleoplasm defined by DNA staining with DAPI. For lentivirus-expressed HBx, the average intensity of cytoplasmic and nuclear fluorescence signals was determined using ImageJ by manually selecting the corresponding areas (based on nuclear staining and cell edges and excluding regions with staining artifacts). For visualization of the spatial localization of HBx and Smc6 within a nucleus, a series of z-stacks was acquired with a step of 200 nm (the pixel size was 50.1 nm), and volumetric rendering was performed using Imaris 9.1.2 by defining the boundaries of the objects and applying the Surfaces function. Images were adjusted equally within each data set for brightness, contrast, and sharpness using PowerPoint 2010 (Microsoft, Redmond, WA, USA).

### Protein stability assay.

PHH were infected with HBV as described previously (13). At day 5 postinfection, 100 μg/ml cycloheximide was added to stop protein translation. The cells were then fixed as described above at various times after cycloheximide addition. The protein signal intensity was measured by confocal imaging and normalized to a signal obtained for an untreated sample fixed simultaneously. The data were plotted as a percentage of the initial signal versus time (hours) after cycloheximide addition and fit to a first-order exponential decay equation {[*A*] = [*A*]_0_*e*^(−*k_t_*)^} using GraphPad Prism version 7.03 (GraphPad Software, San Diego, CA) to calculate the decay constant (−*k*). The half-life (*t*_1/2_) for each protein was calculated in hours using ln 2/*k*.

### Extracellular HBeAg and HBV DNA quantification.

Extracellular HBeAg and HBV DNA levels were measured as previously described (13, 42). HBeAg levels were measured by an electrochemiluminescence assay (Meso Scale Discovery, Rockville, MD). Extracellular HBV DNA was purified from cell culture medium using a DNeasy 96 blood and tissue kit (Qiagen) and quantified by qPCR on a QuantStudio 7 Flex real-time PCR system (Thermo Fisher Scientific), using primers and a probe designed for the HBx region of the HBV genome: forward primer 5′-CCG TCT GTG CCT TCT CAT CTG-3′, reverse primer 5′-AGT CCA AGA GTY CTC TTA TGY AAG ACC TT-3′, and probe 5′-56FAM [5(6)-carboxyfluorescein]-CCG TGT GCA-ZEN-CTT CGC TTC ACC TCT GC-3IABkFQ (Iowa Black fluorescein quencher)-3′. HBV DNA levels were normalized to β-actin.

### HBV sequence analysis.

HBx consensus sequences for genotypes A, B, C, and E were derived from the collection of curated sequences in the HBV database (43). Removing the ∼1% of sequences with atypical insertions or deletions, there were 735 sequences for genotype A, 197 for genotype B, 1,377 for genotype C, and 235 for genotype E. Sequences were first aligned with ClustalW2 (44), and a consensus was generated for each genotype using BioEdit (45). The HBx genotype D sequence was the same as the sequence of HBx expressed in HepAD38 cells (GenBank accession number AUG90794). The HBx sequences are provided in Table 1.

### Statistical analysis.

Data are expressed as means ± standard errors of the means (SEM). Statistical significance was tested using a two-tailed *t* test (for two-sample comparisons) or one-way analysis of variance (ANOVA) with multiple-comparison correction (for multiple comparisons), using GraphPad Prism version 7.03 (GraphPad Software). A *P* value of <0.05 was considered significant.
